# Cellular senescence: when growth stimulation meets cell cycle arrest

**DOI:** 10.18632/aging.204543

**Published:** 2023-02-19

**Authors:** Mikhail V. Blagosklonny

**Affiliations:** 1 Roswell Park Comprehensive Cancer Center, NY 14263, USA

**Keywords:** rapamycin, mTOR, hyperfunction theory of aging, cell volume and enlargement, gerogenic conversion

## Abstract

At the very moment of cell-cycle arrest, the cell is not senescent yet. For several days in cell culture, the arrested cell is acquiring a senescent phenotype. What is happening during this geroconversion? Cellular enlargement (hypertrophy) and hyperfunctions (lysosomal and hyper-secretory) are hallmarks of geroconversion.

## Epigraph

*“Growth stimulation leads to cellular senescence when the cell cycle is blocked”* [1].

## Arrest is not yet senescence

Not anything that causes arrest causes senescence. For example, serum withdrawal, contact inhibition, nutrient starvation and rapamycin cause reversible arrest (quiescence) instead of senescence. What these conditions have in common is that they inhibit cellular mass or volume growth and specifically inhibit the mTOR pathway. (Of note: in the cell culture, quiescent cells will eventually succumb to senescence, because even rapamycin does not suppress geroconversion completely).

To induce senescence, DNA-damaging agents p21 and p16 cause cell-cycle arrest. Freshly arrested cells do not have senescent phenotype. During several days, the arrested cells acquire a large, flat morphology, beta-Gal positivity and Senescence-Associated Secretory Phenotype (SASP) [2–4]. The acquisition of senescent phenotype in arrested cells is known as gerogenic conversion or geroconversion [4–8].

Geroconversion is a continuation of cellular growth, when the cell cycle is blocked [1]. It may also partially occur in proliferating cells and is overstimulated in cell culture conditions. Cellular mass (volume) growth is driven in part by growth-promoting pathways such as mTOR [6]. And this is how the anti-aging activity of rapamycin was predicted, before life-extension was shown in animals [9].

Despite the obvious (acquisition of senescent phenotype takes time via active process), the existence of geroconversion is largely ignored by scientific community. One of the reasons is that in cell culture, geroconversion occurs automatically, unless actively prevented by rapamycin, serum and nutrient withdrawal, contact inhibition, severe hypoxia and some other factors (discussed later). In 2011, it was pointed out that “In cell culture, cell cycle arrest typically leads to senescence, because the cell is overstimulated by serum, nutrients, oncogenes and so on. Therefore, cell cycle arrest is sufficient to cause senescence, especially in cancer cells. This is why arrest of cell cycle is confused with senescence” [10].

## Growth stimulation drives senescence during cell cycle arrest

Nutrients, mitogens or growth factors (GF), hormones (e.g., insulin and testosterone), cytoplasmic oncoproteins, oxygen and other factors stimulate growth-promoting pathways such as mTOR and MAPK, which stimulate both cellular mass growth, cyclin D induction and cell cycle progression. In the absence of growth stimulation (e.g., GF or serum withdrawal), MAPK and mTOR are deactivated. This slows down both cellular mass growth and cell cycle progression, and the cell becomes quiescent. Re-addition of growth factors allows quiescent cells to re-start proliferation [5, 6].

**Figure 1:**
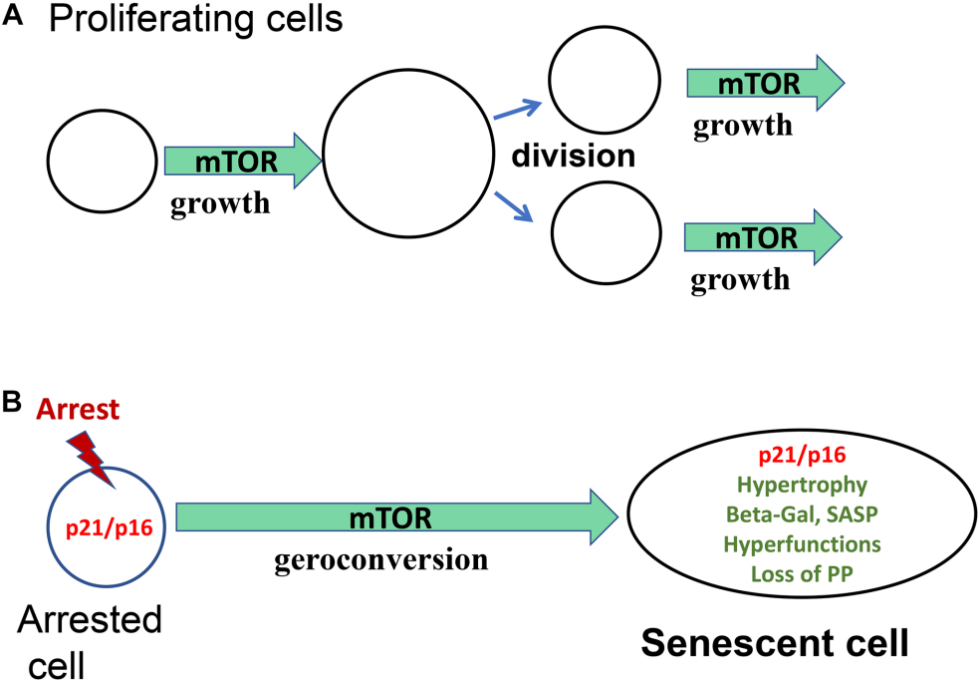
**Geroconversion as a form of growth.** (**A**) Proliferating cells. Cellular enlargement (growth) is followed by cell division. mTOR is shown as one of the drivers of growth. (**B**) Arrested cells. In the arrest cell (p21 and p16) cellular enlargement is followed by cell division. mTOR is shown as one of the drivers of geroconversion.

In proliferating cells, mTOR drives cellular mass growth, and this growth in cell size is balanced by cell division (Figure 1A). In quiescent cells, mTOR is deactivated, and the cell cycle is arrested. What would happen if the cell cycle were arrested, but mTOR is still active?

This condition can be caused by induction of CDK inhibitors (p21 and p16), which block the cell cycle, without affecting growth-promoting pathways such as mTOR and MAPK [6].

When the cell cycle is arrested by p21/p16, then mTOR drives growth in the absence of cell division, causing cellular hypertrophy (a large, flat cell morphology), lysosomal hyperfunction (beta-Gal-staining) and other hyperfunctions such as SASP (Figure 1B). It also increases tissue-specific hyperfunctions [6, 11].

Overactivated mTOR causes compensatory resistance to growth factors and insulin, via the pS6K1/IRS feedback loop [12, 13].

In cell culture, p21 and p16 cause cell-cycle arrest fast, but, at the moment of the arrest, the cells are not yet senescent. During the next 3–5 days, the arrested cells acquire the senescent phenotype [2–4]. This process is called geroconversion.

In a typical cell culture, cells are overstimulated by nutrients, serum and oxygen and grow in low cell density, making mTOR maximally active. For example, DMEM contains 5-fold higher than normal blood levels of glucose, higher than even in diabetic patients [13]. This is why it is sufficient to induce cell cycle arrest to induce senescence, unless mTOR-driven geroconversion is actively suppressed by serum withdrawal and contact inhibition, which deactivate mTOR [5, 14].

## Pseudo-DNA-damage response in senescent cells

Molecular damage is not required for geroconversion (like it is not required for growth). For example, p21 and p16 (CDK inhibitors) and cause cell-cycle arrest without causing DNA damage: p21 and p16 directly bind to CDKs to arrest cell cycle. Then still active mTOR, MAPK and other growth-promoting pathways convert this arrest to senescence (geroconversion).

During geroconversion, overactivated kinases such as ATM phosphorylate H2AX, even in the absence of DNA damage [15]. As suggested by Rybak et al. [16] although DNA double-strand breaks always induce γH2AX, the reverse is not true: γH2AX is not an unequivocal marker of these breaks [16–18].

So, detection of γH2AX indicates that the cell may be senescent but does not indicate that it is necessarily caused by DNA damage. Unfortunately, it is not known to most scientists.

## Acute DNA damage can cause arrest, but it’s not yet senescence

Acute DNA damage by radiation and DNA-damaging drugs activates DNA damage response (DDR). While DNA damage response (DDR) causes cell-cycle arrest, it is growth-promoting pathways such as mTOR that convert this arrest to senescent phenotype. (Figure 1B).

[Note: Life-long, gradual accumulation of DNA damage (accumulation of mutations) does not lead to cell-cycle arrest, but, in contrast, contributes to unlimited proliferation, robustness and immortality in cancer cells].

Once again, acute DNA damage or DDR in proliferating cells can lead to cellular senescence, because proliferating is associated with high activity of growth-promoting pathways necessary for geroconversion. When DDR causes arrest, these growth-promoting pathways drive geroconversion [19]. In serum-starved quiescent cells, mTOR is inactive and DNA damage cannot cause senescence. Growth stimulation with serum then drives geroconversion [19].

In the organism, acute DNA damage, or DDR, can lead to cell senescence by arresting proliferating cells. This is an age-independent cellular senescence that may occur at any age. This is also called non-adaptive cell senescence [20].

In contrast, age-dependent cellular senescence may be driven by life-long hyperfunction of growth-promoting pathways, especially in arrested (post-mitotic) cells.

## Proliferative potential

At first, the freshly arrested cells retain proliferative potential (PP) and can re-start proliferation, if cell-cycle arrest is lifted. Following geroconversion, senescent cells cannot proliferate, even when cell-cycle arrest is lifted. The senescent cell may re-enter the cell cycle but cannot progress further or die in mitosis [2–4]. Loss of PP is a marker of the senescent phenotype, and rapamycin partially prevents loss of PP, as it partially prevents other markers of senescent phenotype such as cell hypertrophy, beta-Gal and SASP. Proliferative* potential* should not be confused with proliferation. For example, rapamycin inhibits proliferation but preserves PP. When p16 and p21 were induced for one day and then switched off, the cells resumed proliferation. If p16 was switched off after six days, cells remained phenotypically senescent and could not restart proliferation [2, 3]. Serum starvation [1, 19, 21] and mTOR inhibitors [1, 4, 22], prevent loss of PP during arrest, caused by switchable p21/p16 and the synthetic CDK4/6 inhibitor Palbociclib (PD0332991).

The irreversibility of cell cycle arrest should not be confused with Loss of PP. For example, Doxorubicin, a DNA-damaging drug, can render cell-cycle arrest irreversible, because doxorubicin cannot be easily washed out from the cell. If arrest is irreversible, it is impossible to know whether the cell retained (or not) the proliferative potential.

## Cell hypertrophy (enlargement) as a marker of senescence

The large senescent morphology is the most noticeable feature of senescence in cell culture [23] and in the organism [24]. And it is not coincidental. Geroconversion is a continuation (quasi-program) of cellular growth [25]. At the beginning of geroconversion in p21-arrested cells, cellular mass (protein per well) is increased exponentially, and then growth becomes linear in p21-arrested cells [26]. In agreement, Neurohr et al. showed that within 9 days after doxorubicin-induced arrest, cell size increased linearly 8-fold [21]. Similarly, linear increase in cell volume was observed during arrest caused by the CDKi Palbociclib, and this increase was completely prevented by serum starvation [21]. Rapamycin partially decreases hypertrophy during cell-cycle arrest caused by either p21 or synthetic CDK inhibitors [4, 26]. Pan-mTOR inhibitors more potently suppressed hypertrophy than rapamycin [27, 28].

Thus, hypertrophy is only partially rapamycin-sensitive [26, 27].

## Excessive cell growth as a marker of geroconversion

Geroconversion can occur not only in arrested but also in proliferating cells, if growth stimulation is excessive. For example, stem cells are small, and their size is increased with aging [29], and excessive growth stimulation drives stem cell geroconversion [7, 8].

It was even suggested that an increase in cell size by itself can cause senescence [21, 29, 30]. According to the geroconversion concept, excessive activation of growth-promoting pathways (MAPK, mTOR, etc.,) drives both excessive growth and other hyperfunctions (SASP, lysosomal hyperfunction (beta-Gal), hyper-differentiation). Furthermore, overactivated MAPK and mTOR pathways may induce p53/p21 and cycle arrest [31]. Following cell-cycle arrest, growth becomes even more excessive. Excessive growth and other manifestations of geroconversion are difficult to dissociate, because the manipulations that decrease growth (serum/nutrient starvation, rapamycin) also block MAPK/mTOR network that drives ALL manifestations together. This may suggest that cell size drives senescence rather than hyperfunctional growth-signaling drives senescence-associated hyprertrophy. As suggested, excessive mitogen/growth-stimulation may lead to hypermitogenic arrest [32] and then full-blown cell senescence [9, 31].

## Geroconversion as terminal differentiation

Geroconversion can also be viewed as hypertrophic differentiation. For example, chondrocytes, responsible for bone growth in length, become hypertrophic and undergo senescence [33–36]. Like geroconvesrsion, terminal differentiation is an active process associated with decrease of proliferative potential [37], possible beta-Gal-positivity [38] as well as hypertrophy [39, 40] and increase of cellular functions, mainly tissue-specific functions. Geroconversion can be called gerogenic differentiation. This topic links the organismal/body growth program, hypertrophic differentiation, and geroconversion as a quasi-program of cellular growth and developmentally programmed cellular senescence [20].

## Developmentally programmed cell senescence

While cell senescence is a quasi-programmed in aging, it may be programed in development [20, 41–45]. During mammalian embryonic development, senescent cells are cleared by macrophages, resulting in tissue remodeling [41].

## Oncogene-induced senescence

Hyper-mitogenic stimulation may trigger cell-cycle arrest and simultaneously promote size growth [32, 46–49].

## How should we define cellular senescence?

Cellular senescence is neither functional decline nor caused by chronic accumulation of molecular damage. In contrast, cellular senescence is characterized by universal hyperfunctions such as SASP plus tissue-specific hyperfunctions (senescent beta-cells as an example). Second, whether accumulation of molecular damages (mutations) lead to cancer, cancer cells tend to be immortal. A common definition of cellular senescence as permanent loss of proliferative potential does not recapitulate the most important features of the senescent phenotype, such as hypertrophy and hyperfunctions (e.g., SASP).

Cell senescence is a proliferation-like state in non-proliferating cells. Growth-promoting pathways, including mTOR and MEK/MAPK, drive both growth and geroconversion. When actual growth is completed, growth-promoting pathways drive cellular senescence (Figure 1). Thus, a program of growth becomes a quasi-program of senescence. (Quasi- means pseudo- or “resembling but not real”). Senescent cells resemble proliferating cells but do not proliferate [5]. As “Growth stimulation leads to cellular senescence when the cell cycle is blocked” the molecular hallmark of senescent cells is presented: high levels of p21/p16, phospho-S6 and cyclin D1 [50]. Cell senescence is associated with constitutive, proliferative-like activity of nutrient-sensing and growth-promoting pathways such as mTOR in non-proliferative (arrested) cells.

David Gems and Carina Kern suggested replacing the term cellular senescence with remodeling activation, and SASP with RASP [20]. The key word is activation. According to hyperfunction theory, cellular senescence (or remodeling activation) can be viewed as hyperactivation, hyperfunction, hypertrophy, hyper-differentiation.

In 2003, I proposed “that simultaneous stimulation of mitogen-activated pathways and downstream inhibition of cyclin-dependent kinases leads, ultimately, to cell senescence” [32]. In other words, senescence occurs when growth stimulation meets cell cycle arrest. In agreement, Rapamycin and other rapalogs (Everolimus and Ridaforolimus), pan-mTOR inhibitors [27, 28] and, to a lesser extent, MEK, PI3K, mdm-2 and S6K inhibitors all slow down geroconversion in mammalian cells [1, 22, 26, 51–55].

Numerous studies further confirmed that mTOR is involved in the senescence phenotype [56–69].

Regardless of whether cellular senescence contributes to organismal aging or not, the geroconversion cell culture model is a prototype of the hyperfunction theory of quasi-programmed aging. The geroconversion model introduces the notion of a quasi-program of growth and hyperfunction. Regardless of mechanistic link (or its absence) between cellular senescence and organismal aging, they are analogies. The same pathways that drive geroconversion are involved in organismal aging and age-related diseases. The same drugs that slow down geroconversion also extend lifespan, as tested in animals so far. Targets of gerostatics (e.g., mTOR, PI3K) are involved in aging of animals from worm to mammals. Therefore, gerostatics are anti-aging drugs. The model of geroconversion is useful to discover anti-aging drugs.

## Organismal aging as quasi-program of developmental growth

Like geroconversion is a continuation of cellular growth, the organismal aging is a continuation of developmental growth (see Figure 1 in reference [70]). Aging is not programmed, it is quasi-programmed. A quasi-program is a purposeless continuation of programs that were not turned off upon their completion. This has been discussed in detail [9, 50, 71–75].

Growth and aging are driven by overlapping signaling pathways. As suggested in 2007, “mTOR stands out because (a) it is a hub in the signaling network, (b) it is conserved from plants to animals (c) its inhibitors, rapamycin (Sirolimus) and everolimus, are clinically available drugs” [76]. To be clinically useful, the hyperfunction theory is mTOR-centric.
